# Alternative Future Vegetation Pathways Reveal Potential Transformations of Western US Ecosystems

**DOI:** 10.1111/gcb.70795

**Published:** 2026-03-09

**Authors:** Tyler J. Hoecker, Kimberley T. Davis, Caitlin Littlefield, Jeffrey Chandler, Sean Parks, Andrew Maguire, Kerry Kemp, Svetlana Yegorova, Solomon Dobrowski

**Affiliations:** ^1^ Vibrant Planet, PBC Truckee California USA; ^2^ Pyrologix LLC Missoula Montana USA; ^3^ University of Montana Missoula Montana USA; ^4^ USDA Forest Service Rocky Mountain Research Station, Missoula Fire Science Laboratory Missoula Montana USA; ^5^ Conservation Science Partners Truckee California USA; ^6^ US Geological Survey Climate Adaptation Science Centers Anchorage Alaska USA; ^7^ USDA Forest Service Region 6 Ecology Program Wenatchee Washington USA; ^8^ Wilfrid Laurier University Waterloo Ontario Canada

**Keywords:** climate adaptation, climate analogs, decision‐support, ecological transformation, resilience, species distribution modeling

## Abstract

Managing ecosystems in an era of rapid change is inherently challenging not only because of uncertainty in future climate but also due to diverse responses of ecosystems to climate. Projections of ecological transformation alongside information about plausible vegetation trajectories can help land managers explore divergent scenarios and consider how modeled outcomes match their observations. Climate‐analog impact models (AIMs) compare environmental information (e.g., vegetation types) between sets of climatically similar locations to infer change and can be used to identify multiple outcomes. We used AIMs to project changes in vegetation across the western United States under a mid‐21st century climate scenario, characterize ecological transformation vulnerability based on projection divergence, and demonstrate how AIMs can inform decision‐making. We projected high or very high vulnerability to ecological transformation across 29% of the western US, nearly 1 M km^2^. Vulnerability varied among vegetation groups; 75% of alpine vegetation had high or very high vulnerability vs. 6% of desert scrub. We estimate that 9% of the study area faces a high likelihood of transformation based on combined measures of vulnerability and projection agreement. Transformation at the vegetation type (*n* = 50) level is projected for 40% (1.4 M km^2^) of the study area, based on primary projections. As vegetation shifts towards types supported by a more arid climate, forested area is expected to contract by 9% and subalpine forests specifically by 54%. Elsewhere, vulnerability is low or trajectories are uncertain, implying opportunities for managers to intervene. Dry forests, for example, could be stabilized through vegetation management and intentional fire use. Our findings suggest likely ecological transformations with significant downstream consequences for ecosystem services and natural resources. They are best used within decision‐making frameworks that draw on multiple lines of evidence including local expertise and complementary knowledge systems.

## Graphical abstract

We used climate‐analog impact models (AIMs) to project changes in vegetation across the western United States under a mid‐21st century climate scenario, characterize ecological transformation vulnerability based on projection divergence, and demonstrate how AIMs can inform decision‐making. We projected high or very high vulnerability to ecological transformation across 29% of the western US, nearly 1 M km^2^. We estimate that 9% of the study area faces a high likelihood of transformation based on combined measures of vulnerability and projection agreement. Elsewhere, vulnerability is low or trajectories are uncertain, implying opportunities for managers to intervene. Our findings are best used within decision‐making frameworks that draw on multiple lines of evidence, including local expertise, to inform climate‐adaptation strategies. Our data are freely accessible via vegetationfutures.org.

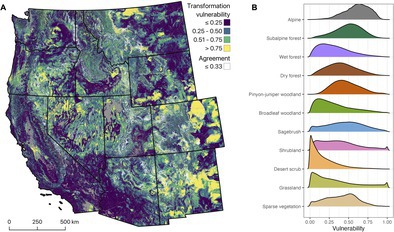

## 1 Introduction

As the pace of climate change accelerates and impacts to natural resources emerge, land managers are faced with a complex challenge: environmental stewardship into an uncertain future. Climate change and disturbances–like severe wildfires, insect outbreaks, drought, resource extraction and land‐clearing–interact to catalyze ecological transformation (Crausbay et al. 2022; Jackson 2021). Disturbances initiate change by removing existing vegetation, and then by altering seed sources and exposing regenerating vegetation to post‐disturbance climates that have diverged from pre‐disturbance conditions (Davis et al. 2019; Moss et al. 2024; Seidl and Turner 2022; Stevens‐Rumann et al. 2018). With ongoing climate change, post‐disturbance conditions are increasingly suitable for a different suite of species than was present prior to disturbance. Paleoecological evidence, recent observations, and future projections agree that climate change and climate‐driven disturbances will transform ecosystems globally, including within the focal region of this study, the western United States (Coop et al. 2020; Hoecker et al. 2023; McDowell et al. 2020). This evidence motivates the development of future ecological scenarios that can guide resource management (Clark‐Wolf et al. 2025). Here, we explore co‐produced projections that can support climate‐change adaptation by revealing multiple plausible vegetation trajectories alongside information about the potential for management intervention to alter course.

Projections of vegetation patterns yield critical insight about ecosystems and their likely trajectories into the future (Guisan et al. 2013). Projections of future vegetation are often based on deterministic models, and divergence among projections under a single climate scenario is not widely embraced as a source of information itself, rather than as a source of uncertainty or error (Clark‐Wolf et al. 2025). Models that project future vegetation are generally not designed to reflect climatic niche overlap among different vegetation types (Broennimann et al. 2012; Parks et al. 2018; Stephenson 1998); instead, they categorically assign one vegetation type to a site based on environmental conditions. Yet, projecting future ecological states is fraught with complexity and multiple outcomes are often plausible. By mitigating the impacts of surprising outcomes and identifying the events, processes, and tipping points that drive change, approaches that embrace variability may better support scenario‐based planning than approaches that focus on a central tendency (Carpenter 2002; Clark‐Wolf et al. 2025).

There are multiple sources of data or process uncertainty in future vegetation projections including model abstractions and assumptions, trends in heat‐trapping gases, input data quality, compounding errors, and more (Thuiller 2004; Wiens et al. 2009). However, plausible variation in projections of vegetation can also arise simply because multiple vegetation types may be supported by the same climatic conditions. Climatic niche overlap can therefore also affect model inference, but should not be conflated with data or process uncertainty because it arises from true biological redundancy among niches (Broennimann et al. 2012). Moreover, dynamics beyond climate contribute to the emergent vegetation type at a given site, including disturbance history, competition, facilitation, species dispersal and migration, disequilibrium dynamics, soil development, biogeographic history, and human activities (Amundson and Jenny 1997; Hastings 1980; Phillips 2007). Consequently, biophysical variability, climatic niche overlap, and chance can together result in variation among plausible projections of vegetation outcomes under climate change. This variation is not necessarily a liability; scientists and natural resource managers can leverage variation in projections to guide planning and action. Scenario‐based decision‐making, in general, and climate adaptation frameworks like resist‐accept‐direct (“RAD”; Schuurman et al. 2020), specifically, help to reconcile divergence among future projections. But, using divergence among projections to inform decision‐making requires that variability is presented in a cogent and actionable way.

Here, we used climate‐analog impact models (AIMs) to project the vegetation patterns that are likely to be supported by reference period (1961–1990) and projected mid‐21st century (+2°C) climates across the western US. These AIMs use vegetation data from locations where the climate is similar to a focal location's reference period or projected mid‐21st century climate to infer the vegetation communities those climates support and detect changes between time periods. AIMs quantify the level of support for different vegetation communities under the same climate, identifying multiple plausible trajectories for vegetation change into the future. We developed a measure of vulnerability to ecological transformation based on the dissimilarity between vegetation communities supported by reference period and mid‐21st century climates, and use the statistical support for a given projection to quantify model certainty. Our specific goals were: (1) project the vegetation types most likely to be supported by reference period and mid‐21st century climates, (2) quantify the vulnerability of reference period vegetation to transformation under mid‐21st century climate, and (3) demonstrate how variability among plausible vegetation projections can guide natural resource management.

## 2 Methods

### 2.1 Climate‐Analog Impact Models (AIMs)

**FIGURE 1:**
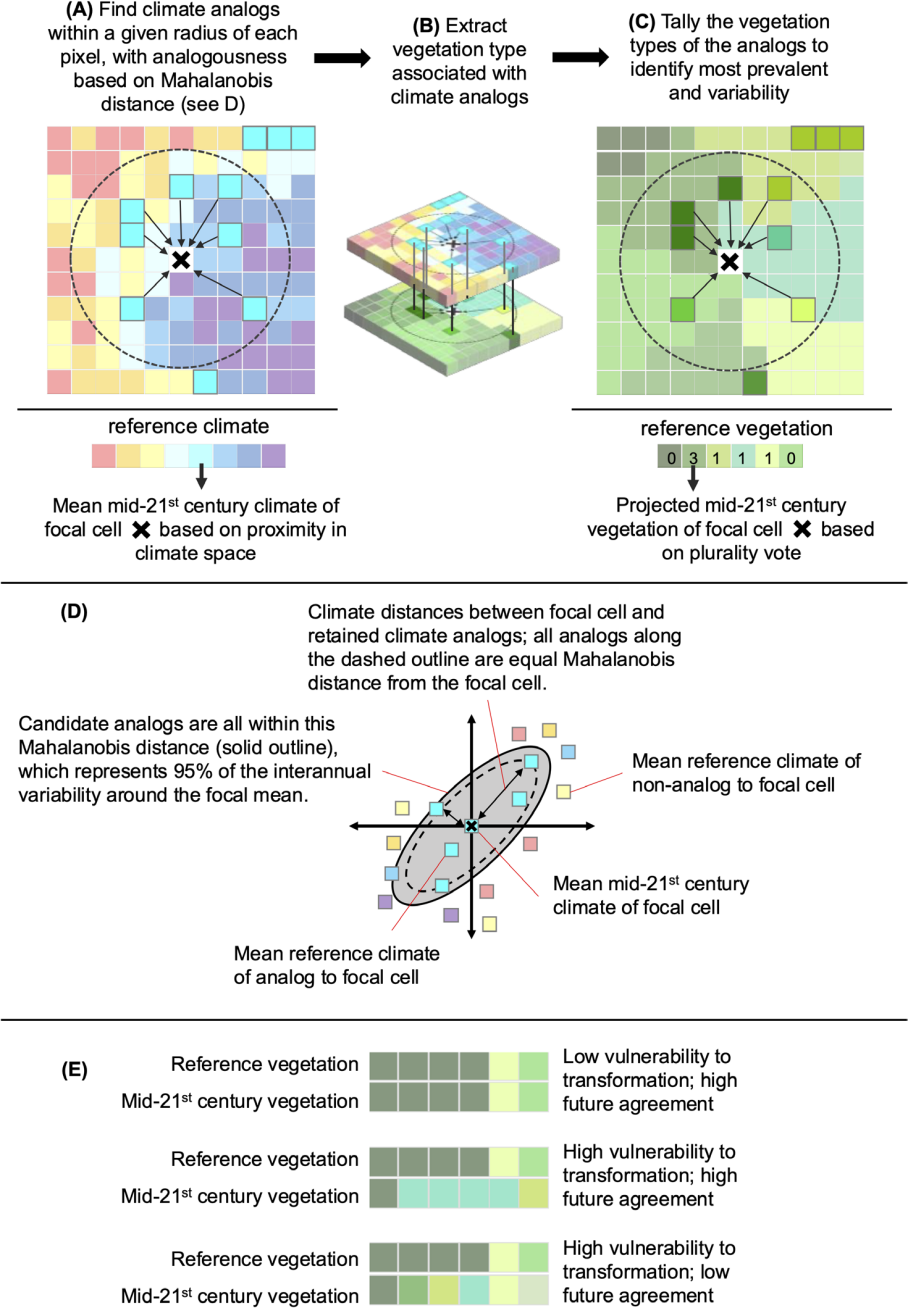
Key steps used to develop climate‐analog impact models of vegetation states and transformation vulnerability. Using downscaled climate data representing the reference period (1961–1990) and mid‐21st century (A), we identified climate analogs within a 500 km radius based on their multivariate climatic similarity to the focal location (D). We identified mid‐21st century (shown) and reference period (not shown) analogs and extracted vegetation information at the analog locations (B). We tallied vegetation among analogs and identified the most prevalent classes (C), and compared the sets of vegetation classes plausibly supported by reference period and mid‐21st century conditions to measure transformation vulnerability and projection agreement (E). Three vulnerability‐agreement examples are shown. Elements of this figure are adapted from Dobrowski et al. (2021) and Yegorova, Dobrowski, Parks, et al. (2025).

We projected the vegetation types supported by reference period and mid‐21st century climates using AIMs (Yegorova, Dobrowski, Parks, et al. 2025). AIMs integrate data‐driven pattern recognition, multivariate statistics, and space‐for‐time substitution to enable projection at scale. AIMs rely on reverse and contemporary climate analogs to project mid‐21st century and reference‐period conditions, respectively. Reverse climate analogs identify locations that, during the reference period, experienced the projected future climate of the focal location. Contemporary analogs identify locations that share reference‐period climate conditions with the focal location. AIMs project ecological states (e.g., dominant vegetation) using observations from climate analogs within a defined geographic distance of a focal location (Figure 1). Because analogs are climatically similar and geographically near, they capture the effects of environmentally and spatially structured ecological processes on vegetation occurrence.

AIMs are a useful alternative to other forms of species distribution modeling because they are spatially explicit and scalable (Yegorova, Dobrowski, Yung, et al. 2025), and AIMs may prove to be more intuitive to interpret than other frameworks. Depending on spatial grain, AIMs can require significant computational costs. However, once a set of analog locations is identified, any data characterizing these locations can be used to draw inference about the focal location, which could be more efficient than developing new models for every ecological response of interest. At scale, AIMs are less computationally intensive than most process‐based simulation models and do not require parameter development or calibration. Like other space‐for‐time‐substitution approaches, AIMs assume that climate is a strong driver of vegetation patterns and that vegetation and climate exist in pseudo‐equilibrium at broad spatiotemporal scales or in aggregate; these assumptions can be violated in some cases, especially at fine scales (Evans et al. 2025).

AIMs implicitly represent spatially autocorrelated latent variables. In other words, because AIMs are constrained to a specified distance around the focal location, and near things tend to be more alike than distant things (Tobler's Law), AIMs are thought to provide more information about underlying processes than non‐spatial models, especially for processes that are difficult or impossible to directly represent (Yegorova, Dobrowski, Yung, et al. 2025). AIMs can be used to make projections for any time period, enhancing the generalizability of the approach. Climate analogs and AIMs are already used to calculate climate velocity (Dobrowski and Parks 2016), measure climate connectivity (Carroll et al. 2018; Littlefield et al. 2017), project changes in vegetation types (Yegorova, Dobrowski, Parks, et al. 2025) and ecosystem services (Povak and Manley 2024), project fire‐regime change (Hoecker et al. 2023; Parks et al. 2018), and project climate impacts to cities and agriculture (Fitzpatrick and Dunn 2019).

### 2.2 Vegetation Data

We used the Biophysical Settings gridded product (30‐m resolution) from LANDFIRE as our source of vegetation data (LANDFIRE 2016), based on input from our land‐management partners. Biophysical Settings (BpS) represent geographically specific groups of species that commonly co‐occur under pseudo‐equilibrium among vegetation, climate, and the historical disturbance regime. BpS data are widely used for natural resource management in the US and, critically for AIMs, are spatially continuous across the study area at fine spatial grain. We aggregated the > 200 BpS classes of the western US into 50 classes (hereafter described as “vegetation *types*”), plus an additional class for barren, rock, or perennial snow and ice cover. We aggregated vegetation *types* again into 13 vegetation *groups* for broad‐scale inference and communication (Table S1); of these, riparian and wetland groups were not modeled, leaving 11 modeled groups. We aggregated classes using hierarchical clustering on climate and fire regime, which we qualitatively reviewed and modified with input from subject matter experts. We focus here on vegetation types and groups, but provide projections at the BpS level in our data repository and note that projections made in the widely adopted BpS system could be cross‐walked to other ecological information. All levels of aggregation (BpS classes, vegetation types, and vegetation groups) were resampled from 30 m to ~220 m resolution following the modal type for alignment with downscaled climate data (next section).

### 2.3 Climate Data

Following previous studies, we identified analogs based on four biologically relevant climate variables (Dobrowski et al. 2021) available from TerraClimate (Abatzoglou et al. 2018): cumulative annual actual evapotranspiration, cumulative annual climatic water deficit (the difference between potential and actual evapotranspiration), mean daily maximum temperature of July, and mean daily minimum temperature of January. Reference period analogs were based on annual and mean climate data for 1961–1990. Mid‐21st century AIMs were based on a scenario characterizing a 2°C increase in global mean surface temperature above the pre‐industrial period (Qin et al. 2020), “+2°C”, which is expected to emerge between 2030 and 2050 (Hausfather 2020). TerraClimate used observed spatiotemporal variability from 1986 to 2015 to produce multi‐model mean monthly projections for 30 individual years representing mid‐21st century climate.

We downscaled annual TerraClimate (Abatzoglou et al. 2018) data from their native ~4 km resolution to ~220 m based on a template of fine‐grained topographic variability in climate using a gradient and inverse‐distance weighting approach (Flint and Flint 2012; Rodman et al. 2020). We used historical data from TopoFire as our template, a high resolution climate data product that integrates station‐based observations and hydrologic simulations to generate continuous surfaces of derived water balance metrics (Holden et al. 2016). We refer to the downscaled climate product as *TopoTerra*, which exhibits high agreement with maximum temperature of July and minimum temperature of January at independent weather stations (*R* ^2^ = 0.94 and 0.95, respectively). TopoTerra data are publicly accessible (https://osf.io/w6jvk) and additional detail on the downscaling method and validation is provided in Hoecker et al. (2025). TopoTerra products improve upon gridded climate datasets that are downscaled using elevational lapse rates by also representing the effects of slope and aspect on insolation and of topographic position on water availability, showing, for example, warmer and drier conditions on south‐facing slopes than on north‐facing slopes.

We identified mid‐21st century climate analogs by calculating the multivariate distance between the mean +2°C climate at each cell and the mean reference period climate of cells within a 500 km radius, then selecting locations nearest to the focal cell in climate space (Figure 1). We measured separation in climate space using Mahalanobis distance, which is equivalent to Euclidean distance in decorrelated PCA space (Mahony et al. 2017). This method uses annual realizations of climate at a focal location to estimate local interannual climate variability and rescales distances to account for multivariate covariance (Figure 1D). We then selected the 100 analogs nearest to the focal pixel in climate space (i.e., lowest Mahalanobis distance). We imposed a 500‐km radius to remove potential analogs from distances that exceed maximum plausible dispersal for many species under similar magnitudes of projected climate change (Chen et al. 2011; Parks et al. 2022; Williams et al. 2007). We identified reference period climate analogs the same way, except dissimilarity was measured between the mean reference period climate at the focal location and the mean reference period climate of potential analogs. We modeled vegetation for the reference period using an AIM instead of using actual vegetation, and then compared reference period AIMs against mid‐21st century AIMs, to ensure our comparisons were based on consistent methods and to prevent misinterpreting the loss of rare types, a known limitation of AIMs. We refer readers to Yegorova, Dobrowski, Parks, et al. (2025) for a thorough treatment of methodological choices during AIM development and their consequences.

### 2.4 AIM‐Based Vegetation Projections

To characterize the range of plausible vegetation types supported by the climate of each focal cell, we extracted vegetation data from the 100 analog locations most similar to the focal location (Figure 1B,C). Applications of AIMs limit the pool of analogs to the 100 most suitable locations because retaining additional analogs provides diminishing information and increases computational costs. We developed a novel approach for identifying a primary, or plurality, vegetation projection while incorporating variability. With each analog considered a “vote” for a vegetation type, we weighted each vote according to its distance from the focal location in climate space, where the nearest analog received a vote approaching 1, the most distant analog received a vote approaching 0, and all others scaled accordingly. The vegetation type with the largest weighted proportion of votes was selected as the ‘primary’ projection; ‘secondary’ and ‘tertiary’ projections were based on types with the second‐ and third‐most support. We evaluated multiple approaches for identifying a primary projection from suitable analogs and found that climate‐weighted voting performed better than unweighted voting. We used the same procedures to build mid‐21st century and reference period AIMs. Finally, we held vegetation types whose occurrences are not strongly related to climate constant between time periods, excluding future change. These included riparian, wetland, and serpentine‐soil types, and we did not model barren or perennially snow‐covered areas. Non‐modeled types covered an area of 297,935 km^2^, leaving a modeling area of 3,401,450 km^2^.

Variation in the vegetation observed at climate analog locations confers a key benefit of AIMs: because every focal location can be compared to a multitude of climatically analogous locations, the vegetation community at each analog location can be used to characterize a range of variation in plausible outcomes and quantify model support for specific projections (Yegorova, Dobrowski, Parks, et al. 2025; Figure 1). We leveraged this capacity to derive two metrics that aid in AIM interpretation: ecological transformation vulnerability and projection agreement. We define an index of ecological transformation vulnerability (hereafter, vulnerability) as the Bray–Curtis dissimilarity (Bray and Curtis 1957) between the 100 observations of vegetation composition plausibly supported by reference period and mid‐21st century climates at a given location (Figure 1E), ranging from 0 to 1. We categorized vulnerability as low (0–0.25), moderate (0.26–0.50), high (0.51–0.75) and very high (> 0.75). We define an index of projection agreement (hereafter, agreement) as the weighted proportions of votes for the mid‐21st century primary projection, ranging from 0 to 1, which we interpret as a measure of model certainty. We categorized agreement into low (0–0.33), moderate (0.34–0.66) and high (> 0.66). We did not interpret agreement for the primary projection among reference‐period analogs; the median value was 0.70 (Figure S1).

### 2.5 Knowledge Co‐Production

Our approach and findings are the result of a participatory knowledge co‐production process among scientists and natural resource managers (Bamzai‐Dodson et al. 2021). Information that is generated by a co‐production process is more likely to be used by practitioners because it is focused on filling specific information gaps that practitioners self‐identify as barriers to action, incorporates knowledge derived from lived experience, and because participation in the process of knowledge production lends credibility and salience to its outcomes (Beier et al. 2017; Cash et al. 2003). To facilitate the integration of our results into decision‐making processes, our key findings are presented in a freely available web‐based tool [vegetationfutures.org]. The tool allows users to generate customized outputs for an area of interest including figures and numerical summaries suitable for inclusion in reports, planning documents, and vulnerability assessments.

Natural resource managers identified the need for fine‐grained spatial projections of future vegetation through a series of engagements facilitated by the author team (Krosby et al. 2020). We further engaged (*sensu* Bamzai‐Dodson et al. 2021) a group of 32 partners from the National Park Service, USDA Forest Service, The Nature Conservancy, the Bureau of Land Management, the Klamath Tribes, and the Washington State Department of Natural Resources (Figure S2) in iterative meetings to guide decisions about data inputs, analyses, and products for the present study. Partners included individuals in roles such as Fire, Forest, and Vegetation Ecologist; Climate Change Coordinator; Forester; Silviculturist; Wildlife Biologist; and Post‐fire Coordinator. Meetings were primarily virtual but also included site visits at National Park Service units. Participant input informed critical decisions including the spatial resolution of climate data, source of vegetation data, vegetation class aggregations, distance constraints on analog selection, metrics associated with projections, decision‐support tool functionality, and final products.

## 3 Results

We evaluated the accuracy of our reference‐period AIM by comparing projections of reference‐period vegetation types to actual vegetation types and to a binary forest or non‐forest classification (Table S2). To understand how the potential for selecting a cluster of near locations around a focal location impacts classification accuracy, we measured the accuracy of reference‐period projections with and without imposing a 25‐km exclusion radius for potential analogs. For vegetation type, Kappa (with exclusion radius) was 0.61 (0.54) and percent correctly classified was 64% (57%); for forest or non‐forest, Kappa was 0.75 (0.72) and percent correctly classified was 90% (89%).

We also examined the distribution of distances to suitable analogs and to projected vegetation types to better understand the model (Figure S3). The mean distance to the closest analog location with the projected future vegetation type was 94 km and 75% of distances were below 122 km. The mean distance to the closest contemporary example of the projected future vegetation type was 3 km.

**FIGURE 2:**
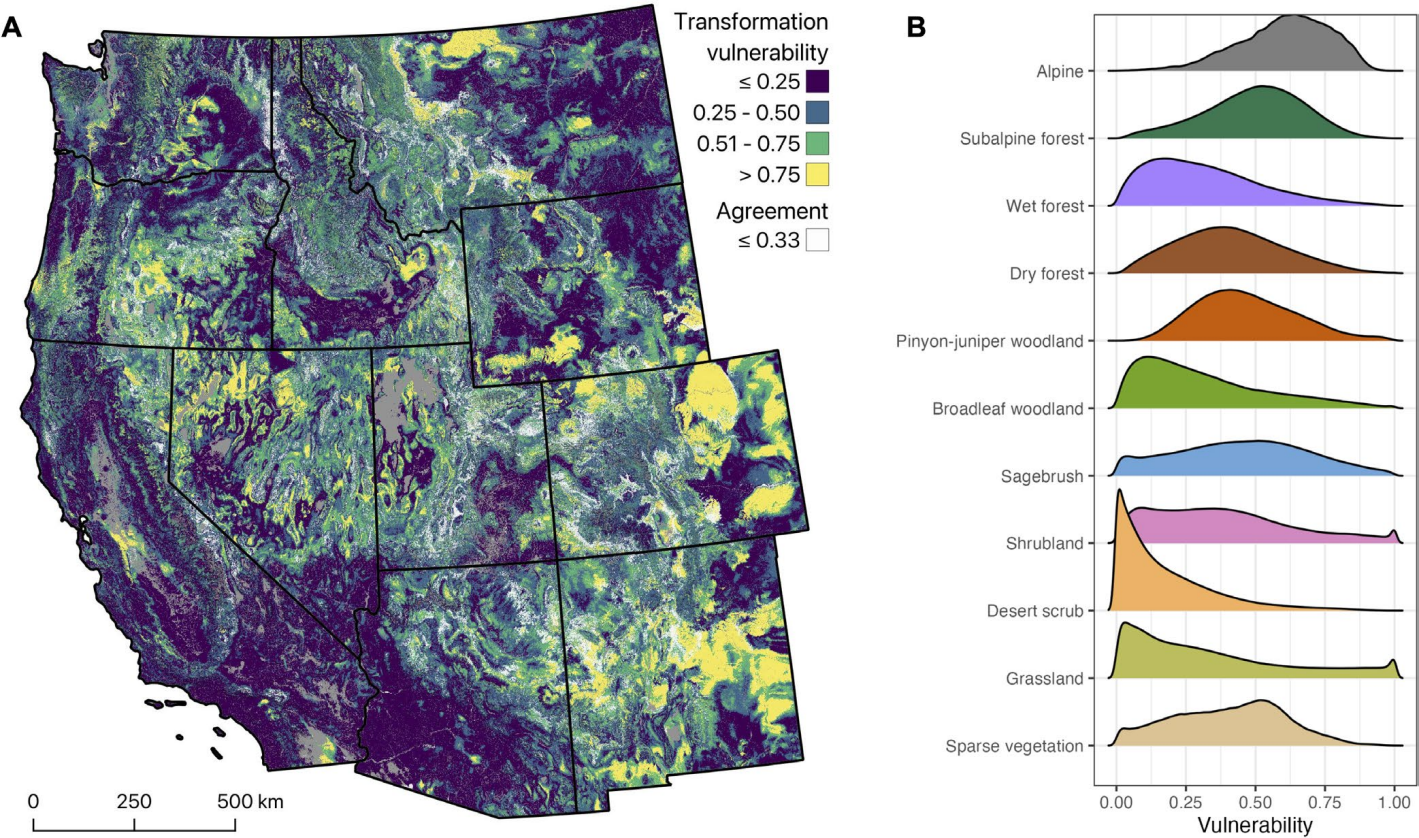
Ecological transformation vulnerability across the western US. (A) Colors indicate vulnerability to vegetation transformation based on the Bray–Curtis dissimilarity index between reference period and mid‐21st century vegetation type composition. Areas with analog agreement ≤ 0.33 are overlaid with white shading. (B) Distributions of vulnerability values by vegetation group.

Our models project widespread vulnerability to transformation in vegetation communities across the western US under mid‐21st century climate (Table 1 and Figure 2). We projected high or very high vulnerability to transformation across 29% of the western US, covering nearly 1 M km^2^ (Table 1). Vulnerability varied among vegetation groups; 75% of alpine vegetation had high or very high vulnerability vs. 6% of desert scrub (Table 1 and Figure 2). Vulnerability was not clearly related to climatic gradients nor to vegetation physiognomy. Vulnerability was generally low for the wet forest, broadleaf woodland, desert scrub, and grassland groups and high or very high for more than a third of the area in the alpine, subalpine forest, pinyon‐juniper woodland, and sagebrush groups (Table 1). Agreement, our measure of projection certainty, also varied considerably across the study area. Agreement was high across 47% of the study area. Agreement varied among vegetation groups (Table 2); 71% of desert scrub had high agreement vs. 11% of pinyon‐juniper woodland. The combination of vulnerability and agreement reveals how projection certainty varies among levels of vulnerability (Table 3). The largest share of the study area, 29%, had low vulnerability and high agreement, whereas 9% of the study area had high or very high vulnerability and high agreement.

**TABLE 1:** Total area and proportions within four levels of transformation vulnerability by vegetation group. See corresponding information mapped in Figure 2.

	**Vulnerability level**			
	Low (≤ 0.25)	Moderate (0.26–0.50)	High (0.51–0.75)	Very high (> 0.75)
	**Area (proportion)** [**km** ^**2**^]			
Total	1,298,190 (0.38)	1,111,985 (0.33)	691,307 (0.20)	302,371 (0.09)
*Vegetation group*				
Sparse vegetation	5007 (0.26)	7601 (0.40)	5704 (0.30)	608 (0.03)
Grassland	552,491 (0.47)	307,401 (0.26)	157,227 (0.13)	150,855 (0.13)
Desert scrub	244,137 (0.75)	63,036 (0.19)	16,294 (0.05)	3817 (0.01)
Shrubland	136,184 (0.35)	138,668 (0.36)	72,227 (0.19)	37,464 (0.10)
Sagebrush	136,788 (0.23)	210,512 (0.35)	191,558 (0.32)	65,556 (0.11)
Broadleaf woodland	51,692 (0.48)	33,386 (0.31)	16,081 (0.15)	5858 (0.05)
Pinyon‐juniper woodland	10,043 (0.11)	46,595 (0.51)	29,593 (0.32)	6014 (0.07)
Dry forest	84,268 (0.22)	181,357 (0.47)	100,942 (0.26)	17,129 (0.04)
Wet forest	58,165 (0.44)	50,289 (0.38)	19,371 (0.15)	4224 (0.03)
Subalpine forest	19,365 (0.11)	72,684 (0.40)	81,186 (0.44)	10,468 (0.06)
Alpine	50 (0.03)	455 (0.23)	1124 (0.56)	379 (0.19)

**TABLE 2:** Total area and proportion of area occupied by vegetation groups during the reference period within three levels of agreement (the weighted proportion of votes for the primary projection). Areas with 0.33 agreement are overlaid with white shading in Figure 2.

	**Analog agreement**		
	Low (≤ 0.33)	Moderate (0.34–0.66)	High (> 0.66)
	**Area (proportion)** [**km** ^**2**^]		
Total	203,129 (0.06)	1,594,658 (0.47)	1,606,067 (0.47)
*Vegetation group*			
Sparse vegetation	258 (0.01)	9604 (0.51)	9056 (0.48)
Grassland	33,368 (0.03)	440,489 (0.38)	694,116 (0.59)
Desert scrub	2032 (0.01)	94,163 (0.29)	231,090 (0.71)
Shrubland	26,311 (0.07)	170,792 (0.44)	187,439 (0.49)
Sagebrush	42,026 (0.07)	312,786 (0.52)	249,602 (0.41)
Broadleaf woodland	3335 (0.03)	57,154 (0.53)	46,528 (0.43)
Pinyon‐juniper woodland	14,726 (0.16)	67,356 (0.73)	10,164 (0.11)
Dry forest	65,975 (0.17)	236,067 (0.62)	81,654 (0.21)
Wet forest	3024 (0.02)	83,240 (0.63)	45,786 (0.35)
Subalpine forest	11,684 (0.06)	121,797 (0.66)	50,222 (0.27)
Alpine	388 (0.19)	1209 (0.60)	411 (0.20)

**TABLE 3:** Summary of area within combinations of transformation vulnerability and analog agreement.

Vulnerability	Agreement			Total
	≤ 0.33	0.34–0.66	> 0.66	
≤ 0.25	11,953 (< 0.01)	296,441 (0.09)	989,796 (0.29)	1,298,190 (0.38)
0.25–0.50	100,607 (0.03)	721,024 (0.21)	290,354 (0.09)	1,111,985 (0.33)
0.51–0.75	80,142 (0.02)	429,906 (0.13)	181,259 (0.05)	691,307 (0.20)
> 0.75	10,426 (< 0.01)	147,287 (0.04)	144,658 (0.04)	302,371 (0.09)
Total	203,129 (0.06)	1,594,658 (0.47)	1,606,067 (0.47)	

*Note:* Color sharing in Total column corresponds to Fig. 2.

**FIGURE 3:**
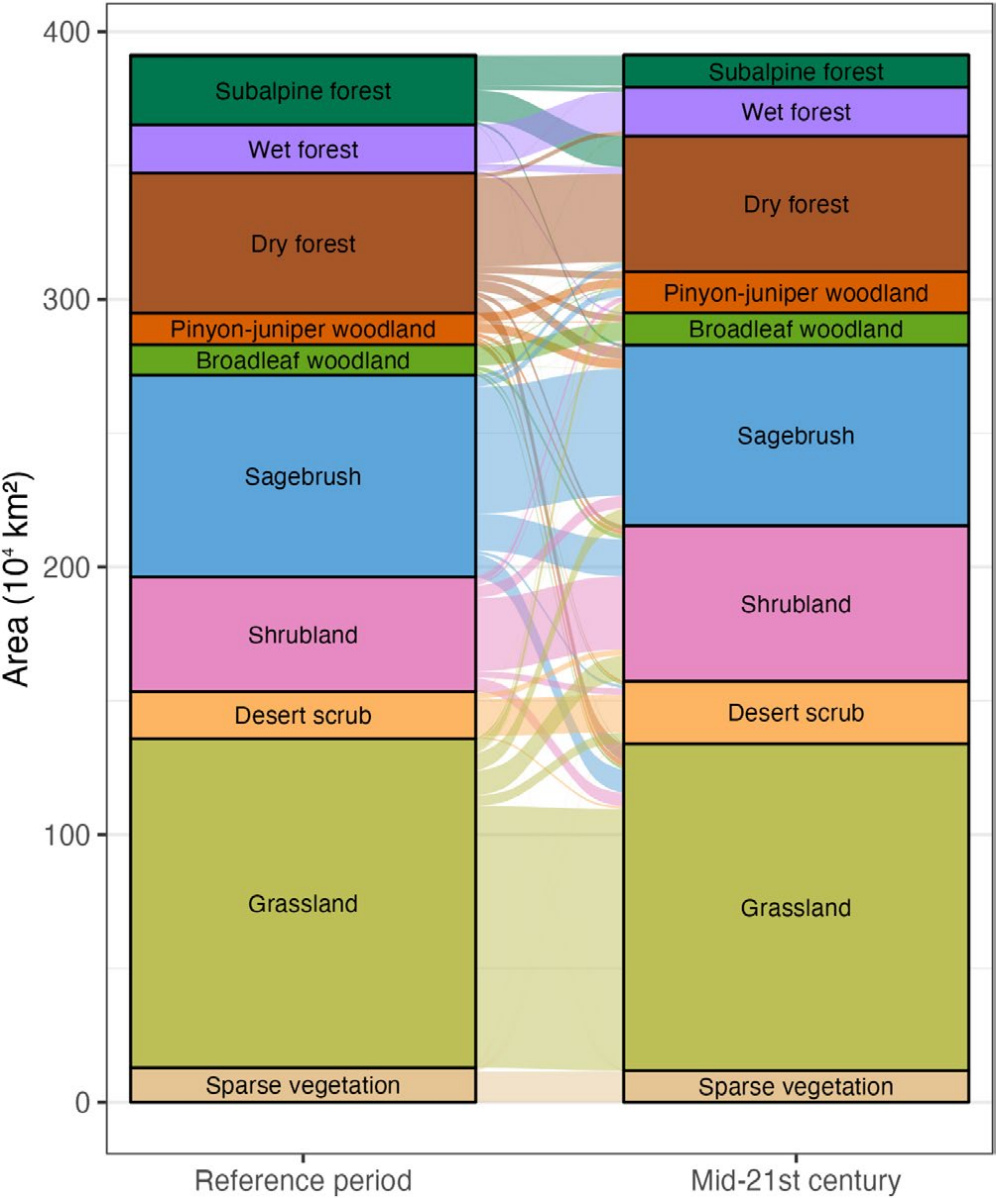
Projected changes in vegetation groups supported by reference period and mid‐21st century climate, based on the primary projection. Due to small areal coverage, the alpine vegetation group label is hidden.

**FIGURE 4:**
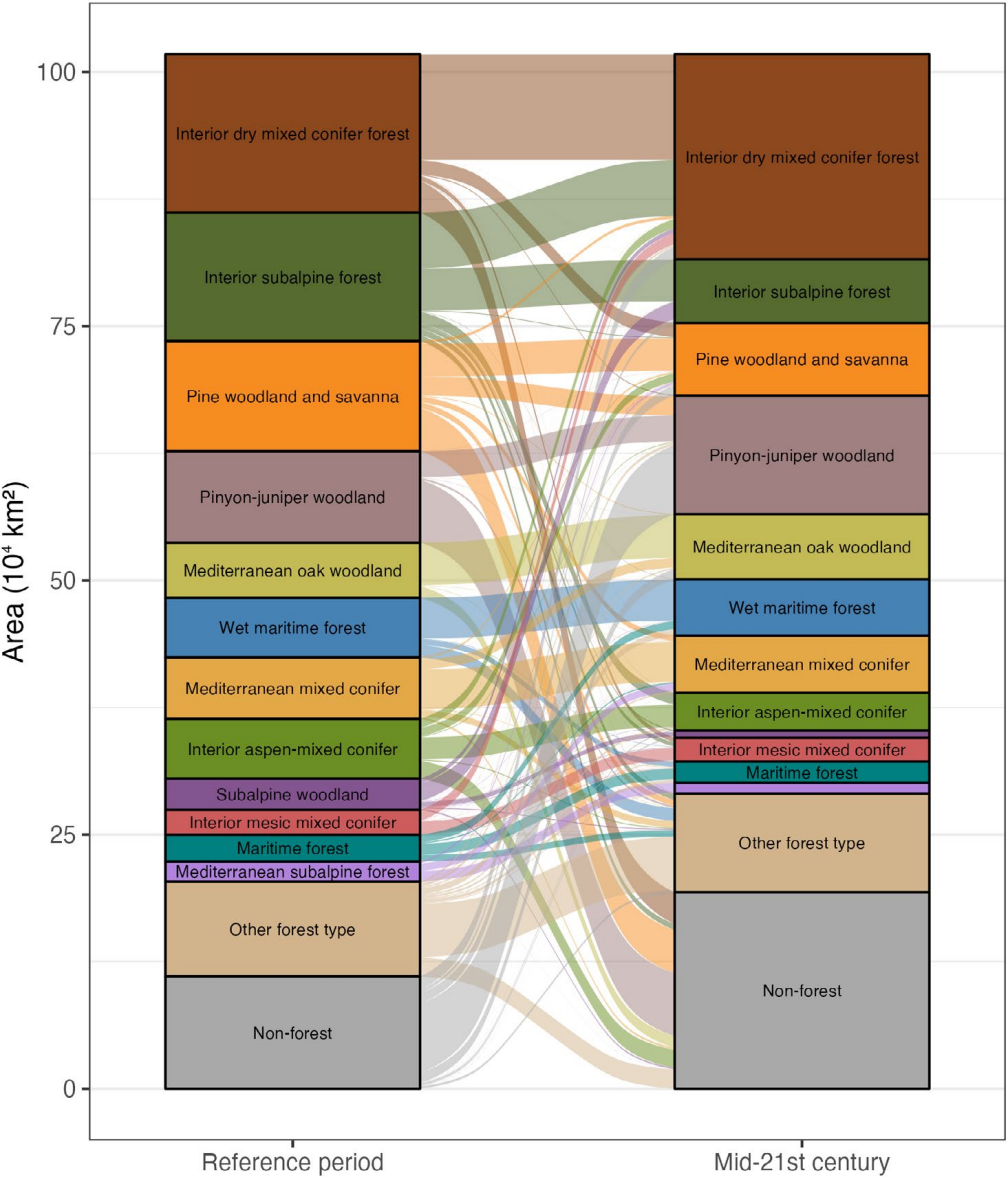
Projected changes in forested vegetation types supported by reference period and mid‐21st century climate, based on the primary analog projection. The “non‐forest” group only includes areas that were projected to be forest under either the reference period or mid‐21st century climate and therefore does not reflect total non‐forested area. See Table S3 for information about the Biophysical Settings included in each vegetation type.

**FIGURE 5:**
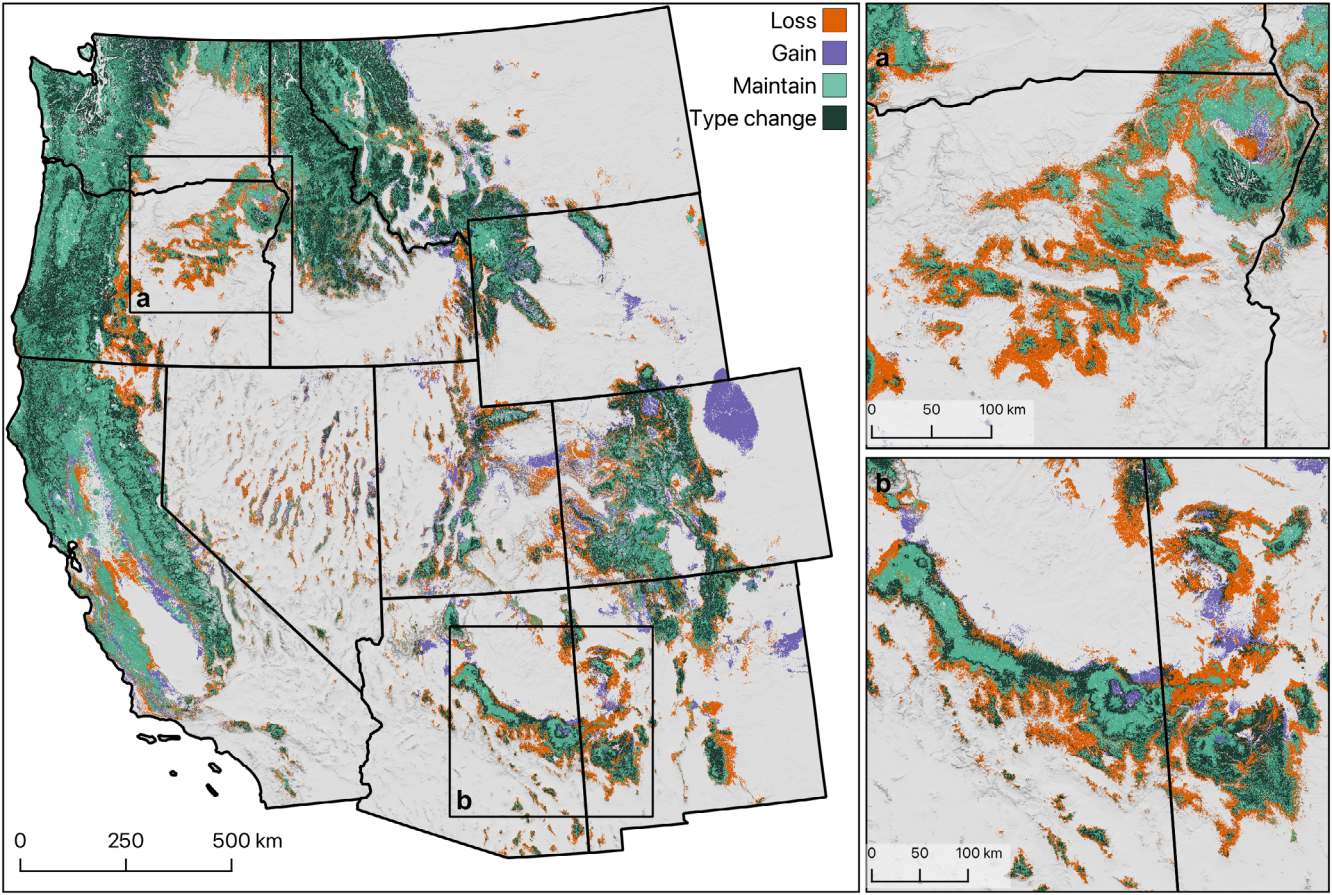
Change in the extent of forested vegetation types. “Loss” indicates areas where the reference‐period climate supported forested types but the mid‐21st century does not, “gain” indicates areas where the mid‐21st century climate supported forested types but the reference‐period climate did not, “maintain” indicates areas where both the reference‐period and mid‐21st century climates support forested types, and “type change” indicates areas where the primary vegetation type projection changed between the reference period and mid‐21st century climate, but both were a forested type.

Results based on primary projections indicate that the relative *proportions* of area suitable for each vegetation group (*n* = 13) remain largely stable, but redistribution of vegetation types was projected for 40% of the study area. The area suitable for subalpine forests is projected to contract while shrublands could expand (Figure 3). Our projections show widespread redistribution of vegetation groups regardless of their relative proportion in either time period (Figure 3). For example, while the proportion of area suitable for pinyon‐juniper remained similar over time, only 14% of locations with a climate currently suitable for pinyon‐juniper retained that suitability under future conditions. Within forested vegetation groups, both the relative proportion and location of areas with climate suitable for each vegetation type are projected to change (Figure 4). We project an expansion of area with climate suitable for interior dry mixed conifer, pinyon‐juniper woodland, and non‐forest types and a contraction for interior subalpine forest, subalpine woodland, and pine woodland and savanna (Figure 5).

The area suitable for subalpine forests is projected to decline by 54% (Figure 3, Table S1 and Figure S4), substantially more than other vegetation groups, with losses concentrated in the forested mountains of the Cascades (Oregon and Washington) and Northern Rockies (Idaho, Montana, Wyoming) and additional areas of loss in the Sierra Nevada (California) and Central Rockies (Colorado, Utah). The magnitude of decline in the subalpine forest group was similarly high based on the secondary and tertiary projections (−60% and −64%, respectively), with much of this area transforming to conditions more suitable for dry mixed conifer forest. Other groups projected to experience a contraction in suitable area include alpine (−100%) and sagebrush (−10%). On the other hand, primary projections suggest the area suitable for pinyon‐juniper woodland and shrubland groups will expand (by 30% and 27%, respectively), with similar or substantially higher increases based on secondary and tertiary projections (Table S1). Although, as noted above, the potential for geographic redistribution of areas suitable for these vegetation groups is high.

At the thematic resolution of vegetation *types* (*n* = 50), we likewise project contractions in the area suitable for subalpine forest and woodland types (> 45% decline; Table S2). However, we also projected the area suitable for pine woodland and savanna to decline (−35%), with much of that area transitioning to climates more suitable for pinyon‐juniper woodlands or non‐forest vegetation types. The loss of area suitable for pine woodland and savanna is concentrated in the southwestern US and the eastern Cascades and Blue Mountains of Oregon (Figure S5) and is not offset by gains in suitable area projected for northern Colorado and southeastern Idaho. However, while the primary projections show a substantial decline in area suitable for pine woodland and savanna, the secondary projections actually show an increase in suitable area of 109%, indicating that pine woodland and savanna are the second most likely vegetation type in many areas. Projected contractions in other widespread vegetation types, such as interior aspen‐mixed conifer, shortgrass prairie, and mixed dwarf sagebrush shrubland, were more consistent across primary, secondary, and tertiary projections (Table S2). Among the most widespread vegetation types, we projected the largest expansion in suitable area for Columbia Plateau grassland (62%), interior dry shrubland (35%), pinyon‐juniper woodland (30%), and interior dry mixed conifer (29%), with some variability among primary, secondary, and tertiary projections (Table S2).

## 4 Discussion

We used a scalable method to project vegetation states supported by mid‐21st century climate and estimate the potential for transformations of vegetation states that are currently supported across the western US. We found that the majority of our study area will experience climates that differ enough from the reference period to support transformations of vegetation groups and types. In some areas, our model projects consequential changes with high confidence, where urgent responses grounded in local management objectives will be needed to avoid undesirable outcomes and where some transformation is likely to be unavoidable. Transformation vulnerability varies widely because climatic niche overlap creates opportunities for landscape‐scale adaptive capacity (Angeler et al. 2019); natural resource managers have opportunities to resist or direct potential changes in places where vulnerability is low or there are multiple plausible outcomes (Lynch et al. 2022; Schuurman et al. 2022).

Our primary vegetation projections suggest the potential for local‐scale transformations in vegetation type across a significant portion, 40%, of the study area, some 1.4 M km^2^. The total extent and relative proportions of most vegetation groups across the western US were projected to remain largely the same—indicating that losses in some areas could be compensated by gains in others. At the same time, geographic redistribution of vegetation communities was common and compensation for range contraction assumes alignment of dispersal processes and landscape configuration with climatic shifts (Årevall et al. 2018). Because redistribution of species will lead to novel assemblages, altered biotic interactions, and changes in ecosystem function, climate‐driven redistribution of vegetation poses a significant risk to ecosystem stability and services (Lawlor et al. 2024; Pecl et al. 2017). Without alignment between environmental conditions and biotic processes, intensifying and more frequent disturbances are likely to catalyze rapid change even as vegetation types like forests, composed of long‐lived species, tolerate periods of disequilibrium between vegetation and climate. As is the case under reference‐period climate, the future climate could plausibly support multiple vegetation types nearly everywhere. Agreement among projections was low for 6% of the study area, suggesting that considering only a primary projection may mischaracterize potential outcomes in these areas.

### 4.1 AIMs Project Uneven Changes Among Vegetation Types

Despite the relative stability in total area that is climatically suitable for vegetation groups, our models project large changes in the area suitable for the finer‐scale vegetation types that comprise groups (Figure 4 and Table S2). For example, forested vegetation types together are projected to contract in extent by 9% (Table S2). Comprehensive interpretation of projected change in each type is beyond the scope of this analysis; below, however, we highlight a selection of changes with important consequences for ecological function and ecosystem service provisioning in the western US. We encourage readers to interrogate changes in vegetation types and areas of interest with the tools available at vegetationfutures.org.

The area with a climate suitable for subalpine forests (including Pacific northwest, Mediterranean, and interior subalpine forests; lodgepole pine forests, and subalpine woodlands) is projected with high agreement to decline by 54% or approximately 106,000 km^2^. Subalpine forests provide important ecosystem services across the mountain West, including the provisioning of water for drinking and agriculture, forest products, habitat for wildlife including dozens of threatened and endangered species, aesthetic qualities, recreational opportunities, Indigenous cultural values, and more. Our finding is consistent with a large body of empirical and simulation‐based evidence for ongoing climate‐driven, fire‐catalyzed loss of subalpine forests in the Greater Yellowstone Ecosystem (Gill et al. 2021; Hansen and Turner 2019; Rammer et al. 2021; Turner et al. 2019), Northern Rocky Mountains (Harvey et al. 2016; Hoecker and Turner 2022; Urza and Sibold 2017), and Southern Rocky Mountains (Andrus et al. 2018). Seed availability from unburned patches, surviving individuals, or serotinous cones is a primary determinant of post‐fire subalpine forest regeneration within climatically suitable areas, suggesting that management activities that promote heterogeneity in spatial patterns of burn severity may be the best opportunities to enhance subalpine forest resilience. However, our models indicate that many areas will likely become climatically unsuitable for subalpine forest regeneration regardless of seed availability. Depending on management goals, responses to these projected changes could include resisting exposure to mortality events, facilitating migration of species adapted to warmer climates, or accepting natural shifts in composition that may include increases in the size or number of unforested patches. In areas where the priority is to maintain forest regardless of species composition, there may be opportunities to direct transformation following disturbances by planting species belonging to the projected vegetation type. For example, many of these areas have high projection agreement that future climate will be suitable for dry forests such that planted dry forest species may successfully establish.

Our model projects a relatively small decline in the area of climate suitable for dry forests (−3.5%), but redistribution of these forests across the landscape and local transformation are common (Figure 5). For example, contraction along the trailing edge of dry forests (Parks et al. 2019) is projected to be offset by expansion of dry forests into subalpine forest types at higher elevation. At the finer resolution of vegetation types within the dry forest group, pine woodland and savanna vegetation types are projected to contract by 35%. This finding is consistent with observations from the northern Rocky Mountains and across the West (Coop 2023; Coop et al. 2020; Davis et al. 2023; Donato et al. 2016; Kemp et al. 2019; Rodman et al. 2020, 2022; Stevens‐Rumann et al. 2018). Our model highlights the potential for multiple trajectories in areas of the Southwest where the reference‐period climate is suitable for ponderosa pine and oak woodlands, supporting evidence from Coop (2023) that transformations to ruderal grasses, bunchgrasses, desert, and gambel‐oak shrublands are all plausible. In these settings, management interventions that reduce fire intensity and thus burn severity will be important to support the persistence of forests in places that remain climatically suitable but are highly exposed to frequent fire activity (Davis et al. 2023; Hoecker et al. 2023).

Our results project a 30% expansion of the area suitable for pinyon‐juniper forests and woodlands across the West (Table 1). However, only 14% of reference‐period pinyon‐juniper forests and woodlands area is projected to remain within a climate suitable for this vegetation type, consistent with recent work highlighting declines in pinyon pine and juniper populations due to high temperatures and drought (Shriver et al. 2022). Our projections that some pinyon‐juniper will convert to shrubland and grassland, while some grasslands and shrublands will convert to pinyon‐juniper, together with low agreement for these projections, imply that a set of interacting processes including disturbance regimes, grazing, edaphic conditions, and invasion biology drive the dynamics of this system alongside climate (Figure 3). Projections of pinyon‐juniper expansion are consistent with the well‐documented encroachment of juniper woodlands into sagebrush and grasslands (Weisberg et al. 2007; Filippelli et al. 2020). But our projections, which are based only on climate and geographic proximity, may over‐predict the future extent of pinyon‐juniper woodlands, and it may be possible to resist or direct these transformations based on management goals (Noel et al. 2025). Similarly, our model projects that shrublands, including mesquite woodlands, semi‐desert chaparral, and shrub‐steppes, will expand by 27%. The increase in shrublands comes primarily from transformations of sagebrush, grassland, and desert scrub vegetation types, which could be interpreted as evidence for primarily climate‐driven change. These transformations could erode habitat for a number of threatened or endangered species, including sage‐grouse and prairie‐chicken (Olsen et al. 2021). Without restoration, continued encroachment of woodlands and shrublands into systems that have been dominated by non‐woody vegetation during the reference period will be enabled by the mid‐21st century climate (Mozelewski et al. 2024).

### 4.2 Using AIM‐Derived Metrics to Guide Disturbance Planning and Response

The observation of low or moderate vulnerability for 71% of the study area provides evidence that management activities that enhance ecosystem resilience to disturbances and support landscape‐scale adaptive capacity (Angeler et al. 2019) could be effective for avoiding undesirable transformations. Strategies to enhance ecosystem resilience include maintaining functional diversity and ecosystem feedback mechanisms, monitoring and directing systems away from irreversible tipping points, protecting ecological memory and material legacies, and learning through low‐stakes experimentation (Allen et al. 2011, 2016; Johnstone et al. 2016). Quantifying differences in transformation vulnerability among sites, as we did, could help managers prepare for and respond to planned and unplanned disturbances. For example, participants in the Harney County Wildfire Collaborative in northeastern Oregon, USA, used a strategic risk assessment framework that integrated pre‐fire planning, incident response, and post‐fire rehabilitation activities to manage complex interactions between fire and invasive annual grasses. The collaborative identified ecologically intact areas where fire use could prevent invasion of annual grasses, transitional areas where fires could catalyze invasion, and degraded areas where restoration of native perennial grasses would be challenging (Wollstein et al. 2022).

Low vulnerability to vegetation transformation based on climatic similarity does not necessarily correlate with low exposure to disturbance, and exposure to abrupt changes in disturbance regimes can initiate ecological transformation despite low apparent climate vulnerability. For example, some of the pinyon‐juniper and dry forests that our model characterizes as low vulnerability are predicted by similar methods to face significant exposure to shifts in fire‐regime attributes (Hoecker et al. 2023). Repeated disturbances in close succession or rapid shifts in disturbance severity can overwhelm forest resilience by undermining fire‐adaptive traits that support post‐fire seed supply (Coop et al. 2020; Gill et al. 2022; Hoecker and Turner 2022; Johnstone et al. 2016). Our model projects low vulnerability for much of California (Figure 2), but, as is widely documented, California faces high exposure to extreme fire and drought events, which can initiate ecological transformation through burn severities that exceed historical norms (Abatzoglou et al. 2021; Agne et al. 2022; Moss et al. 2024; Steel et al. 2022; Stephenson et al. 2024; Stevens et al. 2017; Syphard et al. 2022; Williams et al. 2013). These interpretations suggest that how resource managers prepare for and respond to catalyzing events, which include planned and unplanned disturbances and chronic ecological stress, may be as important as the overall magnitude of climate change in determining future ecosystem states.

In contexts where low predictability precludes anticipatory intervention, maintaining system‐resilience to disturbance (Seidl 2014) and considering alternative future scenarios (Clark‐Wolf et al. 2025) provide two complementary management approaches. We consider predictability low in places where agreement among analogs for the primary projection was less than 0.33 (areas shaded white in Figure 2). For example, in dry fire‐prone forests with low agreement (i.e., multiple plausible trajectories), resilience‐enhancing activities could focus on managing stand density to minimize competition and maintain stable fire‐vegetation dynamics (North et al. 2022; Prichard et al. 2021). Combinations of mechanical biomass removal and intentional fire use, which move systems away from tipping points and maintain key feedback mechanisms, are proving especially effective at enhancing resilience (Davis et al. 2023; Povak and Manley 2024). In addition to enhancing dry forest resilience, considering each of the plausible future vegetation types in areas of low agreement provides the type of information needed for robust scenario planning (Clark‐Wolf et al. 2025). Building on the dry forest example described above, where our models provide evidence that transformations of dry forest to several different forest and non‐forest types are plausible, managers may be able to use their place‐based expertise to weigh the likelihood and consequences of each scenario and choose a management response accordingly. The placed‐based knowledge that managers use to make such decisions is often not the type of information that could be consistently and accurately incorporated into broad‐scale projections, therefore preserving this information is valuable. We encourage caution in the application of these results for decision‐making, particularly at the pixel and vegetation type levels. The dominant trends in transformation vulnerability projected for areas of interest and for higher levels of organization (vegetation groups and forest vs. non‐forest) are less sensitive to uncertainty than pixel‐level projections.

### 4.3 AIMs Carry Important Assumptions

Our results should be interpreted with important assumptions and limitations in mind. AIMs project vegetation change based on similarity in a limited set of climatic variables; we did not perform an exhaustive exploration of potential variables or estimate their relative importance. Likewise, AIM modeling relies on a number of methodological choices including the number of potential and suitable analogs considered, minimum and maximum distances to analogs, method for consolidating projections among analogs, the source of vegetation data and its limitations, and the extent and spatial resolution of analysis. We explored model sensitivity to some of these choices (see Methods) but could not evaluate all of them. Like many other species distribution modeling approaches, AIMs project the climatic suitability for particular vegetation types, but do not project vegetation types per se, as they do not represent processes of disturbance of existing types (see Discussion for extensive treatment), dispersal, or establishment of new types. Thus, our results should be paired with information about key biological processes that would enable or constrain the establishment of the vegetation types projected by AIMs. The geographic constraints built into AIMs should better represent non‐climatic processes than unconstrained approaches. Although analogs could be identified up to 500 km away, the distances to plurality types, upon which projections are based, are generally much shorter (most < 100 km). On the reference period landscape, both the nearest analog representing the plurality type and the nearest examples of projected future types are shorter yet (most < 10 km). Together these results suggest that dispersal of the projected types is plausible in most cases. By building on and refining our approach through continued quantitative testing of methodological decisions, other research groups can improve this method and its applicability to a broader range of problems. These assumptions notwithstanding, our results underscore that land managers will continue to grapple with vegetation transformation across the western US as climate change and intensifying disturbances unfold. Leveraging the variation in plausible future vegetation types provides one pathway for navigating such transformation.

Revealing alternative outcomes and variability in evidence–as we have done here–adds complexity to model interpretation, but it also provides nuance that supports place‐based decision‐making drawing on multiple lines of evidence and complementary knowledge systems (Bonebrake et al. 2018). We present our findings with a perspective that complexity is inherent to adaptation planning and that embracing uncertainty can lead to better management responses.

## Author Contributions

**Tyler J. Hoecker:** conceptualization, methodology, software, formal analysis, investigation, data curation, writing – original draft, visualization, supervision. **Kimberley T. Davis:** conceptualization, methodology, formal analysis, investigation, data curation, writing – review and editing, project administration, funding acquisition. **Caitlin Littlefield:** conceptualization, methodology, investigation, visualization, writing – review and editing, project administration. **Jeffrey Chandler:** software, formal analysis, data curation, writing – review and editing, methodology, validation. **Sean Parks:** conceptualization, methodology, writing – review and editing. **Andrew Maguire:** methodology, data curation, writing – reviewing and editing, software, visualization. **Svetlana Yegorova:** methodology, writing – review and editing. **Kerry Kemp:** conceptualization, writing – review and editing. **Solomon Dobrowski:** conceptualization, methodology, investigation, writing – review and editing, resources, supervision, project administration, funding acquisition.

## Funding

This work was supported by the Northwest Climate Adaptation Science Center, G23AC00072‐00.

## Conflicts of Interest

The authors declare no conflicts of interest.

## Supporting information


**Data S1:** gcb70795‐sup‐0001‐DataS1.pdf.

## Data Availability

Data and code used in this analysis are available via an Open Science Foundation archive [https://osf.io/4pu5x] and are associated with https://doi.org/10.17605/OSF.IO/4PU5X. TopoTerra climate data are available via an Open Science Foundation arhive [https://osf.io/w6jvk/]. Results of this analysis can also be explored and accessed at vegetationfutures.org.
