# Supplementary material for: Alternative Future Vegetation Pathways Reveal Potential Transformations of Western US Ecosystems
Source: Glob Chang Biol. 2026 Mar 9;32(3):e70795. doi: 10.1111/gcb.70795 (PMC12969547; doi:10.1111/gcb.70795)
Supplement: Supplementary file 1 — Data S1: gcb70795‐sup‐0001‐DataS1.pdf. [file GCB-32-e70795-s001.pdf]

Supplementary Information to Hoecker et al. 2026: Alternative future vegetation pathways reveal potential transformations of western US ecosystems

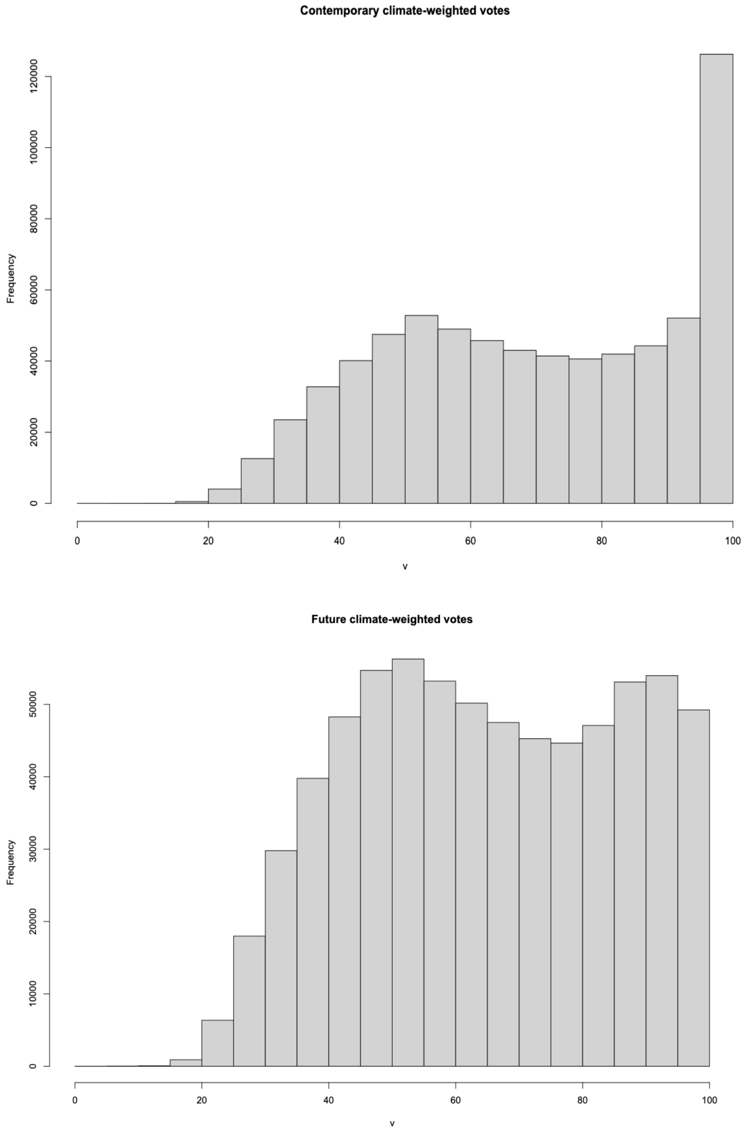

Figure 1. Distribution of weighted votes for reference period (top) and mid-21st century primary projections (bottom). To calculate the weighted vote for a vegetation type, we first rescaled the climatic similarity of each analog to 0-1 (such that the most similar analog gets a vote = 1 and the least similar gets a vote approaching 0) and then summed the weighted votes for each vegetation type.

Table 1. Grouping of Biophysical Settings into intermediate and broad thematic scale groups. Groupings were based on quantitative information about climate and species composition and expert opinion about groupings appropriate in a natural resource management context.

See file Multi-Level Vegetation Classification for AIMs

Table 2. Total projected extent of broad vegetation groups supported by reference period and future climate. The primary (1st), secondary (2nd), and tertiary (3rd) vegetation projections are shown. Parenthetical values indicate the percent change relative to the reference period. The distributions of riparian and wetland vegetation types are not primarily driven by climate and were held constant.

	**Area [10^4 km^2] (Percent change from reference period)**			
**Vegetation type**	Reference period	Mid-21st century - Primary	Mid-21st century - Secondary	Mid-21st century - Tertiary
Dry forest	40.3	38.9 (-3.5)	46.5 (15.4)	44.2 (9.7)
Wet forest	14.7	15 (2)	17.1 (16.3)	13.5 (-8.2)
Broadleaf woodland	10.5	10.9 (3.8)	11.7 (11.4)	9.3 (-11.4)
Pinyon-juniper woodland	9	11.7 (30)	21.3 (136.7)	24.7 (174.4)
Subalpine forest and woodland	19.8	9.2 (-53.5)	8 (-59.6)	7.1 (-64.1)
Shrubland	37.9	48.3 (27.4)	67.5 (78.1)	50.4 (33)
Sagebrush	63.1	57 (-9.7)	63.2 (0.2)	56.5 (-10.5)
Desert scrub	30.8	36.6 (18.8)	19.7 (-36)	9.9 (-67.9)
Grassland	121.1	120.6 (-0.4)	57.2 (-52.8)	53 (-56.2)
Sparse vegetation	10.5	9.5 (-9.5)	35 (233.3)	28.2 (168.6)
Alpine	0.2	0 (-100)	0.3 (50)	0.9 (350)
Riparian	15.3	-	-	-
Wetlands	6.7	-	-	-

Table 3. Total projected extent of vegetation types under reference period and mid-21st century climate. The primary, second, and tertiary mid-21st century projections are shown. Parenthetical values indicate the percent change relative to the reference period.

	**Area [10^4 km^2] (Percent change from reference period)**			
**Vegetation type**	**Reference period**	**Future - 1st**	**Future - 2nd**	**Future - 3rd**
Barren	8.6	8.6 (0)	8.6 (0)	8.6 (0)
Desert	1.4	0.8 (-42.9)	23.6 (1585.7)	16.8 (1100)
Warm desert grassland	12.8	13.6 (6.2)	12.2 (-4.7)	6.8 (-46.9)
Interior dry grassland	3.1	1.7 (-45.2)	4.6 (48.4)	6.7 (116.1)
Columbia Plateau grassland	7.6	12.3 (61.8)	8.1 (6.6)	7.5 (-1.3)
Interior montane-subalpine grassland	2.4	1.7 (-29.2)	4.2 (75)	6.7 (179.2)
Coastal grassland	0.4	0.7 (75)	1.6 (300)	2.7 (575)
Shortgrass prairie	26.4	16.4 (-37.9)	11.2 (-57.6)	13.2 (-50)
Mixedgrass prairie	68.4	74.3 (8.6)	10.6 (-84.5)	7.2 (-89.5)
Tallgrass prairie	0	0 (0)	4.7 (NA)	2.2 (NA)
Warm desert scrub	30.2	35.8 (18.5)	19.5 (-35.4)	9.6 (-68.2)
Tamaulipan savanna and scrub	0.6	0.7 (16.7)	0.2 (-66.7)	0.3 (-50)
Great Plains dry shrubland	5.4	7.3 (35.2)	26.9 (398.1)	12.9 (138.9)
Interior dry shrubland	26.2	35.4 (35.1)	24 (-8.4)	18.6 (-29)
Big sagebrush shrubland	1.3	0.5 (-61.5)	2.2 (69.2)	4 (207.7)
Mixed dwarf sagebrush shrubland	11.5	3.6 (-68.7)	9.3 (-19.1)	11.9 (3.5)
Basin and Wyoming big sagebrush	36.8	41.9 (13.9)	37.4 (1.6)	25 (-32.1)
Mountain big sagebrush	13.4	11 (-17.9)	14.3 (6.7)	15.6 (16.4)
Chaparral	4	3.9 (-2.5)	6.8 (70)	5.5 (37.5)
Montane shrubland	1	0.5 (-50)	6.8 (580)	9.4 (840)

Gambel oak shrubland	1.2	1.2 (0)	3 (150)	4 (233.3)
Southern woodland and savanna	0.9	0.1 (-88.9)	0.6 (-33.3)	0.3 (-66.7)
Southwestern oak woodland	1.4	1.1 (-21.4)	1.4 (0)	3 (114.3)
Mediterranean oak woodland	6.9	7.8 (13)	6.3 (-8.7)	3 (-56.5)
Pacific Northwest oak savanna	1.2	1.7 (41.7)	0.5 (-58.3)	0.8 (-33.3)
Pinyon-juniper woodland	9	11.7 (30)	21.3 (136.7)	24.7 (174.4)
Interior broadleaf woodland	0.1	0.2 (100)	2.9 (2800)	2.2 (2100)
Interior aspen-mixed conifer	5.9	3.7 (-37.3)	4.1 (-30.5)	5.1 (-13.6)
Pine woodland and savanna	11	7.2 (-34.5)	23 (109.1)	24.2 (120)
Mediterranean mixed conifer	6.2	5.8 (-6.5)	2.8 (-54.8)	2.8 (-54.8)
Mediterranean and PNW mixed evergreen	1.2	1.6 (33.3)	2.2 (83.3)	1.9 (58.3)
Mediterranean coastal mixed evergreen	0.4	0.4 (0)	0.9 (125)	0.8 (100)
Serpentine mixed conifer	0.3	0.3 (0)	0.3 (0)	0.3 (0)
Interior dry mixed conifer forest	15.8	20.4 (29.1)	14.1 (-10.8)	9.9 (-37.3)
Interior mesic mixed conifer	2.7	2.8 (3.7)	5 (85.2)	3.4 (25.9)
Redwood forest	1.2	2.1 (75)	1 (-16.7)	0.8 (-33.3)
Maritime forest	2.7	2.1 (-22.2)	4.3 (59.3)	3.2 (18.5)
Wet maritime forest	6.2	5.9 (-4.8)	3.5 (-43.5)	1.9 (-69.4)
Cedar-hemlock forest	0.3	0.6 (100)	1.9 (533.3)	2.2 (633.3)
PNW subalpine forest	0.9	0.3 (-66.7)	0.4 (-55.6)	0.5 (-44.4)
Hypermaritime forest	1.1	1.1 (0)	0.6 (-45.5)	1.1 (0)
Interior lodgepole pine	1	0.8 (-20)	1.1 (10)	1.3 (30)
Interior subalpine forest	12.9	6.3 (-51.2)	4.7 (-63.6)	3.9 (-69.8)

Mediterranean subalpine forest	2	1.1 (-45)	0.5 (-75)	0.5 (-75)
Subalpine woodland	3.1	0.7 (-77.4)	1.4 (-54.8)	0.9 (-71)
Subalpine & alpine non-forest	0.2	0 (-100)	0.3 (50)	0.9 (350)
Sparse montane	0.5	0.1 (-80)	0.2 (-60)	0.4 (-20)
Wetlands	6.7	6.7 (0)	6.7 (0)	6.7 (0)
Interior riparian	11.5	11.5 (0)	11.5 (0)	11.5 (0)
Pacific riparian systems	3.8	3.8 (0)	3.8 (0)	3.8 (0)
Sparse grassland	0	0 (0)	2.5 (NA)	2.4 (NA)

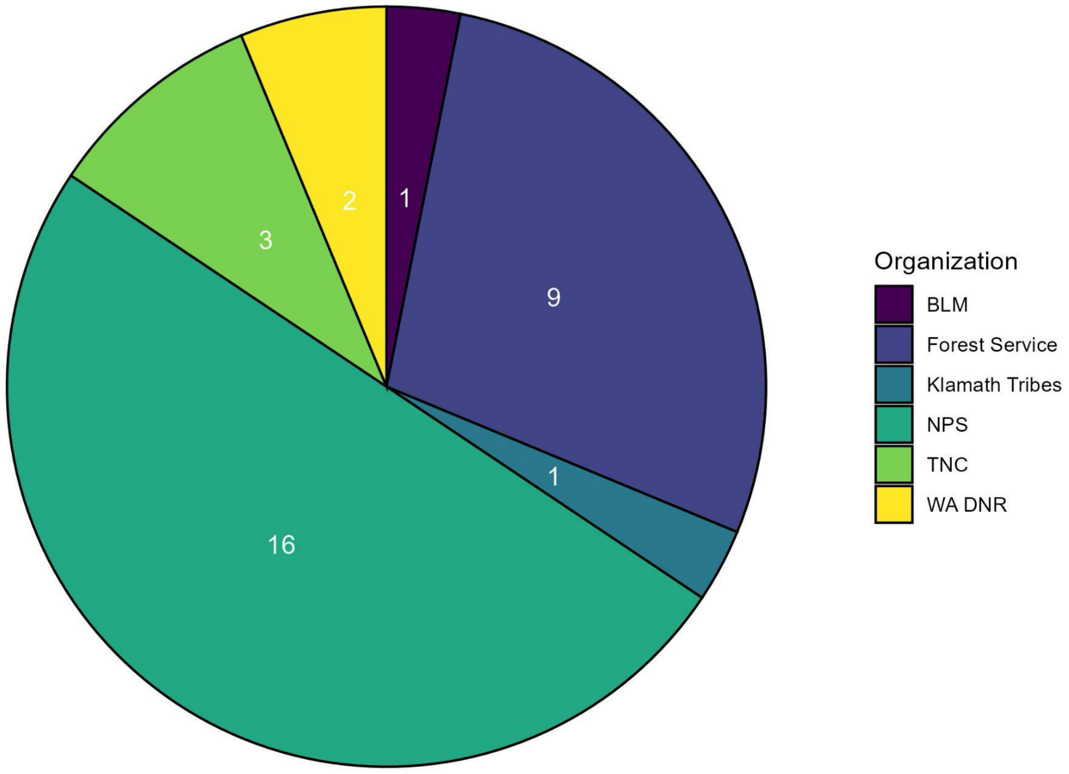

Figure 2. Number and affiliation of partners who participated in one or more co-production meetings.

Table 2. Validation metrics for reference period projections of vegetation type and forest vs non-forest. An exclusion radius of zero was used in the present analysis.

Prediction	Percent correctly classified	Cohen's Kappa	Exclusion radius (km)
Vegetation type	64	0.61	0
Forest vs non-forest	90	0.75	0
Vegetation type	57	0.54	25
Forest vs non-forest	89	0.72	25

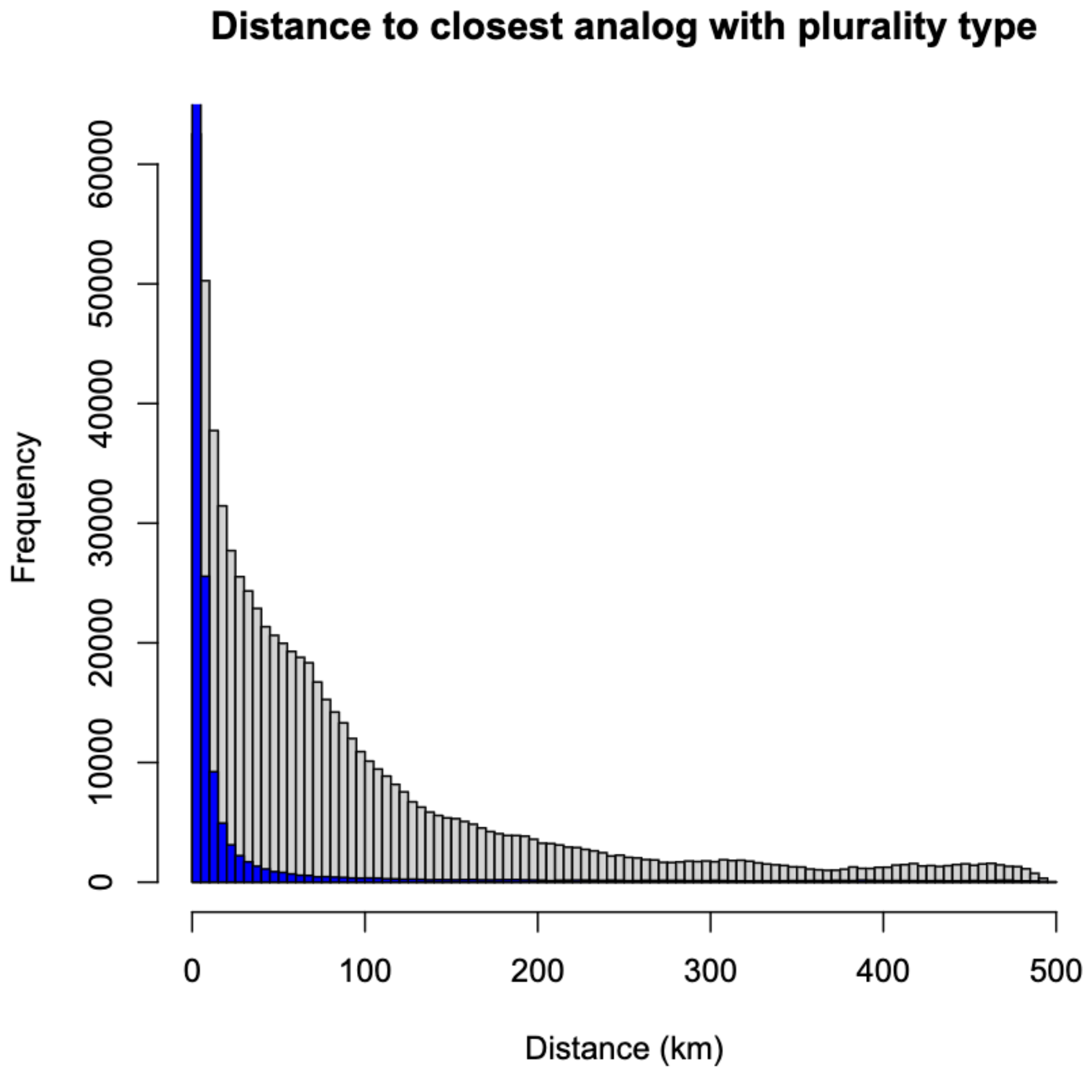

Figure 3. Distribution of distances to the closest analog location with the vegetation type represented by the majority of suitable analogs, for reference period analogs (blue) and future analogs (grey).

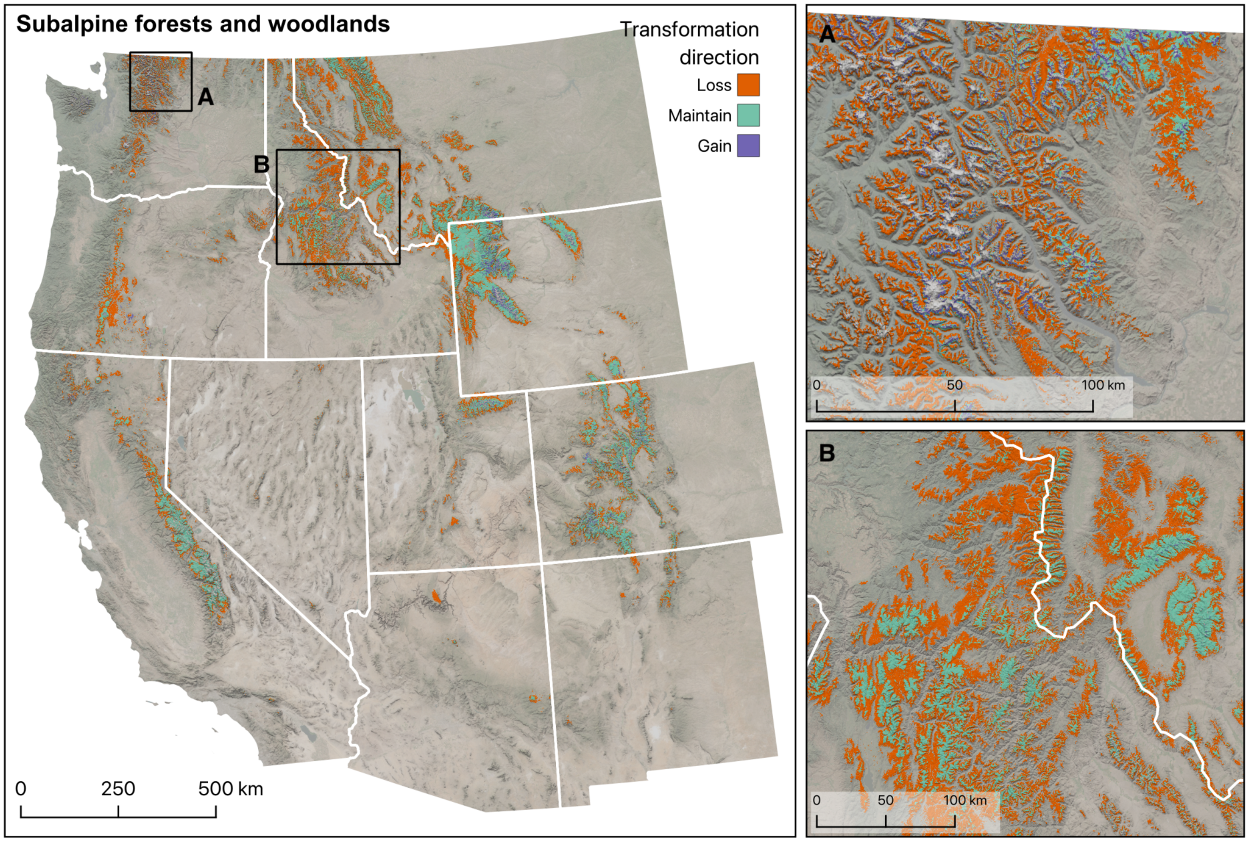

Figure 4. Projected changes in the distribution of the subalpine forest group. The subalpine forest group includes Pacific northwest subalpine forest, subalpine forest, interior lodgepole pine forest, Mediterranean subalpine forest, and subalpine woodland vegetation types.

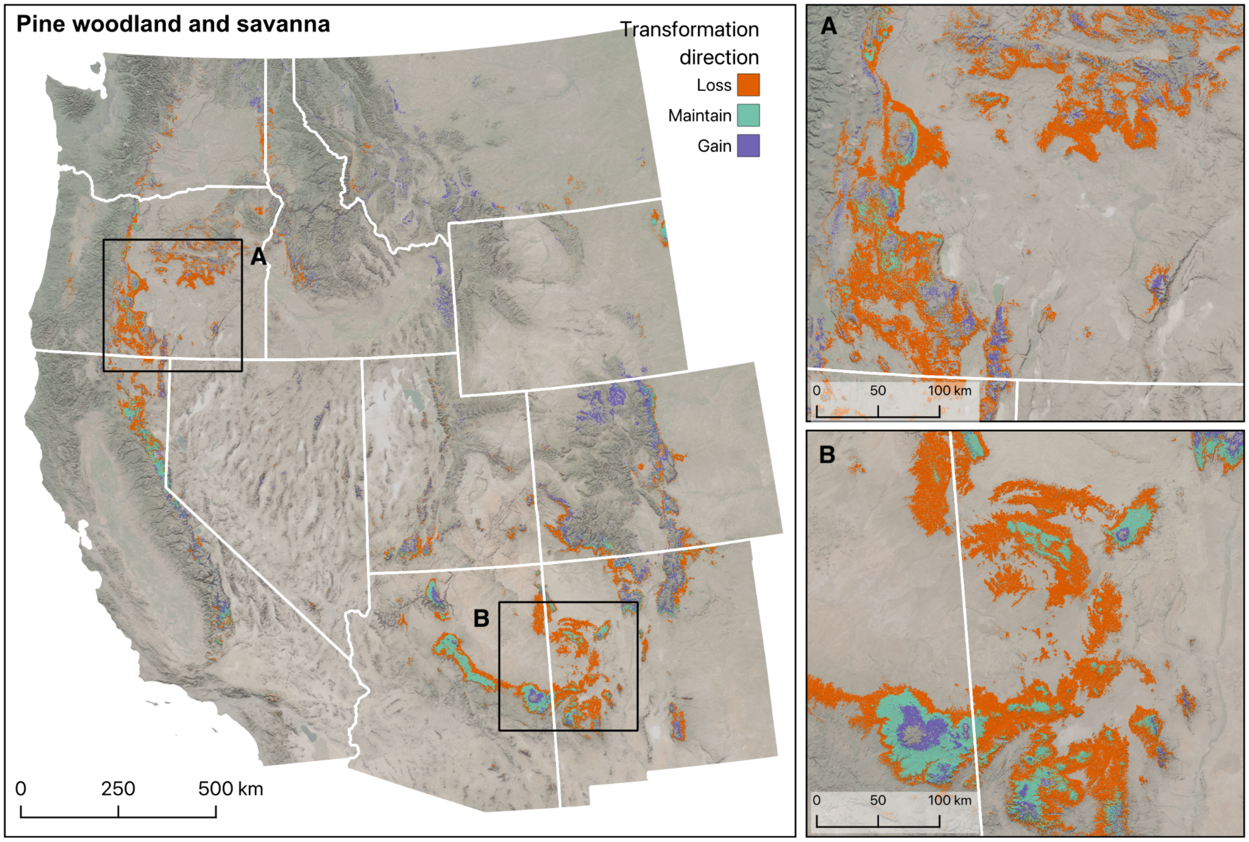

Figure 5. Projected changes in the distribution of the pine woodland and savanna vegetation type (which is part of the broader "dry forest" group) based on the primary projection.
